# Translating research findings into practice – the implementation of kangaroo mother care in Ghana

**DOI:** 10.1186/1748-5908-7-75

**Published:** 2012-08-13

**Authors:** Anne-Marie Bergh, Rhoda Manu, Karen Davy, Elise van Rooyen, Gloria Quansah Asare, J Koku Awoonor Williams, McDamien Dedzo, Akwasi Twumasi, Alexis Nang-beifubah

**Affiliations:** 1 MRC Unit for Maternal and Infant Health Care Strategies, University of Pretoria, Private Bag X323, Arcadia 0007, Pretoria, South Africa; 2 UNICEF Ghana, P. O. Box AN 5051, Accra, Ghana; 3 Family Health, Ghana Health Service, Accra, Ghana; 4 Ghana Health Service, Upper East Region, Bolgatanga, Ghana; 5 Ghana Health Service, Central Region, Cape Coast, Ghana; 6 Ghana Health Service, Upper West Region, Wa, Ghana; 7 Ghana Health Service, Northern Region, Tamale, Ghana

**Keywords:** Infant care, Premature infant, Program evaluation, Ghana, Kangaroo mother care

## Abstract

**Background:**

Kangaroo mother care (KMC) is a safe and effective method of caring for low birth weight infants and is promoted for its potential to improve newborn survival. Many countries find it difficult to take KMC to scale in healthcare facilities providing newborn care. *KMC Ghana* was an initiative to scale up KMC in four regions in Ghana. Research findings from two outreach trials in South Africa informed the design of the initiative. Two key points of departure were to equip healthcare facilities that conduct deliveries with the necessary skills for KMC practice and to single out KMC for special attention instead of embedding it in other newborn care initiatives. This paper describes the contextualisation and practical application of previous research findings and the results of monitoring the progress of the implementation of KMC in Ghana.

**Methods:**

A three-phase outreach intervention was adapted from previous research findings to suit the local setting. A more structured system of KMC regional steering committees was introduced to drive the process and take the initiative forward. During Phase I, health workers in regions and districts were oriented in KMC and received basic support for the management of the outreach. Phase II entailed the strengthening of the regional steering committees. Phase III comprised a more formal assessment, utilising a previously validated KMC progress-monitoring instrument.

**Results:**

Twenty-six out of 38 hospitals (68 %) scored over 10 out of 30 and had reached the level of ‘evidence of practice’ by the end of Phase III. Seven hospitals exceeded expected performance by scoring at the level of ‘evidence of routine and institutionalised practice.’ The collective mean score for all participating hospitals was 12.07. Hospitals that had attained baby-friendly status or had been re-accredited in the five years before the intervention scored significantly better than the rest, with a mean score of 14.64.

**Conclusion:**

The *KMC Ghana* initiative demonstrated how research findings regarding successful outreach for the implementation of KMC could be transferred to a different context by making context-appropriate adaptations to the model.

## Background

Kangaroo mother care (KMC) is a simple and low-cost method developed for the care of low birth weight (LBW) and premature neonates. The newborn is bound in an upright position—skin-to-skin—against the mother’s chest. Other components of KMC include exclusive breastfeeding where possible and early discharge from hospital, with appropriate follow-up care [1]. KMC can be practised in any situation or context, because no special equipment or technology such as cots, heaters or incubators is needed. There is evidence that KMC helps with thermal regulation, reduces stress, protects against infection, enhances breastfeeding, lactation, and bonding, improves infant growth, and contributes to improved neonatal survival [2-4]. Since the introduction of KMC more than 30 years ago in Bogotá Colombia, different forms of KMC practice have spread across the world. In the 1980s and 1990s, KMC was introduced in African countries such as Ethiopia [5], Mozambique [6], South Africa [7], and Zimbabwe [8,9], mostly in teaching hospitals with no further scale-up to lower levels of care.

Reducing neonatal mortality rates is one of the focal areas for achieving Millennium Development Goal 4, which relates to child survival [10]. At the time of the implementation of KMC, Ghana’s under-five mortality rate (U5MR) was estimated at between 111 and 115 deaths per 1,000 live births [11-13] and the infant mortality rate (IMR) at between 71 and 73 deaths per 1,000 live births [12,13]. Although the U5MR and the IMR decreased over the previous decade in Ghana, the neonatal mortality rate (NMR) remained static at 43 deaths per 1,000 live births [11,13,14]. The three main causes of death were infection (32 %), prematurity (26 %), and asphyxia (23 %) [14]. Estimates of the LBW rate ranged from 9.1 % [12] to 16 % [14]. These small infants accounted for the majority of newborn deaths. KMC was therefore identified as one of the solutions to the problems associated with high neonatal mortality rates in Ghana.

The Ghana Health Service (GHS) operates the public health system, which is decentralised into 10 regions. The *KMC Ghana* initiative was a collaborative project run by GHS in four regions, which comprised a total of 51 districts with 38 hospitals. The project was funded by the United Nations Children’s Fund (UNICEF) and received technical support from the South African Medical Research Council’s Maternal and Infant Health Care Strategies Unit and the University of Pretoria. The design of *KMC Ghana* was based on previous experience and research results on appropriate outreach strategies for the implementation and scale-up of KMC. In two randomised trials, the use of a multimedia implementation package combined with an introductory workshop and two or three sessions of face-to-face facilitation was found to be successful in scaling up KMC in three provinces in South Africa [15,16]. In one of the trials, it was found that the site of facilitation, either on site or at a centre of excellence, did not influence the ability of a hospital to implement KMC, and it was recommended that the choice of outreach strategy should be guided by local circumstances, cost, and the availability of skilled facilitators [16].

The aim of this paper is to describe the processes followed in the translation of the findings from implementing KMC in South Africa to a contextualised, practical application in Ghana and the results of the monitoring of the progress made with the implementation of KMC.

## Methods

### Points of departure

Although a large proportion of births still take place at home, the point of departure of *KMC Ghana* was that KMC should first be established and practised with infants delivered at healthcare facilities in the project regions. A long-term goal was the establishment of regional hospitals as KMC centres of excellence for providing future training. Twenty-four hour, continuous KMC services were to be established at district hospitals that could become reference points from which the practice could be extended to the care of LBW infants delivered at home—about one-half of infants were born at home at the time when the initiative was implemented [11,12]. Because a study on community-based KMC in Bangladesh did not demonstrate a reduction in neonatal or infant mortality [17], a cautious approach was adopted to community KMC, as it was essential to first sufficiently strengthen KMC services in healthcare facilities before expanding the programme [4,18,19]. Although the main focus was on introducing KMC in district and regional hospitals, other community healthcare centres and community organisations were targeted for sensitisation and information purposes, because not all districts had a hospital.

*KMC Ghana* differed from approaches embedding KMC in general newborn care or LBW care training or those following the pattern of national or regional adaptation of materials, followed by the training of master trainers. The difficulties associated with the so-called cascade or train-the-trainer model as a top-down process [20] are well known. For example, the information and misinformation may be transmitted instead of creating opportunities for experiential, collaborative, and reflective learning, and the trainers and master trainers chosen may not be based at grass roots and may not be involved in the day-to-day practicalities of a particular practice or procedure that they have to teach. Sloan *et al*. [21] also caution against the use of unqualified or inexperienced KMC trainers in the formal classroom situation. Furthermore, the dilution effect of a train-the-trainer model as the information is cascaded down has the potential to allow the basic messages to become distorted [20,22-24]—‘less and less is understood the further one goes down the cascade’ [25].

The introduction of KMC as a new method of care is often embedded in other newborn care training packages. Our prior experience has shown that where KMC was part of a comprehensive newborn package it often ‘got lost’ when it came to implementation. A ‘pedestal’ approach was therefore followed, in which KMC was targeted for special attention until it was well established and healthcare workers had had sufficient time to make the paradigm shift to integrate KMC into the continuum of newborn care. The planning for training in *KMC Ghana* was also informed by findings from a previous evaluation of KMC in Malawi regarding the unfeasibility of taking healthcare workers out of the workplace for prolonged periods of time; a more practical recommendation of a two- to three-day orientation and training programme was made [26].

### The intervention

The South African model of outreach for implementing KMC was not replicated in *KMC Ghana* because of differences in context, available resources, and the needs identified during the initial situational analysis. No local experience of KMC existed at the time of the implementation, and on-site facilitation at each hospital was not feasible. The project partners therefore considered a different kind of longitudinal, ‘open door’ approach that emphasised a gradual ‘immersion’ into KMC to be more appropriate, allowing for a strong reliance on the local health workers to facilitate this process of immersion at grass roots.

Furthermore, a multi-disciplinary, collaborative team approach to KMC implementation and education was used in the intervention. A Ghanaian project manager who is a medical doctor and three international facilitators with backgrounds in health systems research, neonatology, and nursing education and management, respectively, coordinated the initiative in collaboration with the GHS regional KMC steering committees. One of the facilitators was resident in Ghana when the initiative was planned, and the other two paid a familiarisation visit to the country, held deliberations with key role-players at the national and regional levels of the health system, and visited selected healthcare facilities to gain a better understanding of potential challenges that were likely to arise when importing an outreach model from elsewhere.

**Figure 1:**
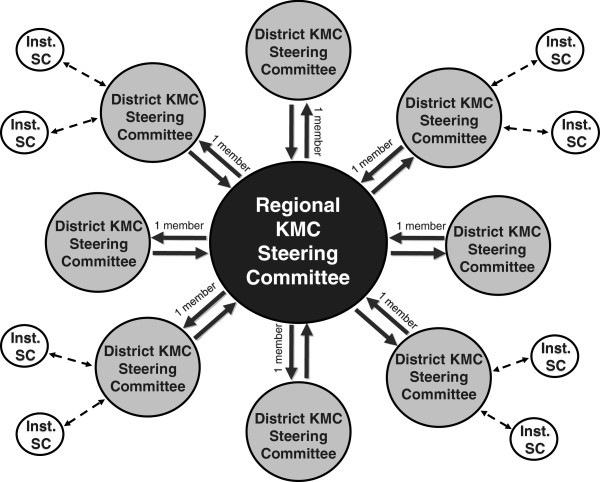
KMC Steering Committee activities.

The notion of using steering committees (SCs) to drive and supervise the implementation process was a key factor in *KMC Ghana*. This was formalised in regional KMC SCs that included a representative from each district. Districts were responsible for the actual implementation actions. In some areas, district and institutional steering committees were also formed (see Figure 1). The regional SCs met three to four times during the first year of implementation to discuss progress and plan further actions.

Technical support for the steering committees was provided in three phases. This process is summarised in Table 1. The aim of Phase I was to help regions and districts to get acquainted with KMC and to provide them with basic support for the management of the outreach. Phase II entailed the strengthening of the regional SCs. In Phase III, each hospital was visited for a more formal assessment of progress with KMC implementation. Although the three phases of points of contact between facilitators and health service representatives were part of the deliverables in the donor contract, the local activities and processes in-between were flexible and were shaped to the emerging needs of a healthcare facility, district, or region, with local participants getting their own feedback and solving their own problems as they occurred.

**Table 1:** Phases in the scale-up of KMC in the project regions

**Month**	**Phase**	**Date**	**Activities per region**
	Preparation	2007/2008	· Situation analysis in each region
			· Introduction of facilitators to key national and regional role-players
			· Familiarisation of facilitators with health system and conditions on the ground
0	I	May/June 2008	· Two-day introductory workshop – three delegates per district
			· One-day steering committee workshop (management of implementation) – one delegate per district
6	II	Nov 2008	· Two-day advanced steering committee workshop – one delegate per district
12	III	May 2009	· Two-day progress-monitoring workshop – selected steering committee members in each region
			· Field monitoring of progress in KMC implementation
			· One-to-two-day debriefing and report-writing workshop

The outreach commenced with six two-day introductory workshops in the four project regions where a multidisciplinary team of three representatives per district was oriented in the basic knowledge and skills needed to practise skin-to-skin care and implement KMC. Each workshop had two overarching themes: the basics of KMC (knowledge of components, benefits, and the application of KMC practice) and the principles that underpin a workable implementation plan. Training materials included a KMC implementation workbook [27], a poster, examples of guidelines and job aids, a KMC DVD or video, and a KMC wrap (for tying a baby in the skin-to-skin position).

At the introductory workshops one delegate per district was nominated to serve on the regional SC, and they attended an additional one-day workshop for orientation in the management of KMC implementation at different levels. The focus was on a planned, yet individualised approach to implementing KMC that could help make it possible to achieve sustainable KMC practice. The implementation workbook was used as a reference tool and participants received further examples of practice standards, management regimes, guidelines, and policies specifically for contextual adaptation. Assignments were given to the SCs to be completed in preparation for the advanced workshop in Phase II.

Six months later, the regional SC members attended one of four advanced capacity-building workshops to report back on progress within the region, expand KMC practice knowledge, discuss KMC implementation challenges, and continue developing facilitation, management, problem-solving and critical-thinking skills. These workshops were planned around the unique clinical and management gaps identified by participants during the first six months of KMC implementation. The identified gaps were incorporated in the clinical goals set for these workshops: record keeping; revision of KMC practice and main components (position, nutrition, follow-up); and provision of additional clinical knowledge and skills in these areas, with a special focus on the feeding of the LBW infant, the handling of preterm infants, and communication. Implementation goals revolved around the following: witnessing and expressing the achievements and challenges of each region’s districts; preparing the region and the districts for the progress monitoring that was to follow in six months; developing district and regional action plans for the way forward; providing guidance and assistance with problems and challenges; and providing further supportive materials.

The approach in all the workshops was the development of ownership, accountability, and responsibility for one’s own learning and actions. Facilitation approaches to promote openness and effective communication included interactive participation, collaborative team work, and experiential and reflective learning. These were accompanied by the development of detailed action plans by individuals or districts that included all the activities to be performed, as well as the deadlines and responsible persons pertaining to each activity. Most plans of action included the debriefing of the district health management team and hospital management, different forms of sensitisation and orientation to a variety of target groups in hospitals and health centres, as well as health workers in the community (*e.g.*, traditional birth attendants and community-based surveillance volunteers). Most healthcare facilities also investigated the creation of some form of KMC space such as a dedicated KMC room or a special corner within the maternity section. Action plans were seen as evolving instruments that could be modified in response to changing circumstances.

One of the ‘hallmarks’ of *KMC Ghana* was the provision of instant feedback after each contact opportunity. Tools and materials developed during workshops were collated, so that at the conclusion of each workshop all materials owned by the workshop participants were immediately accessible for use. Every regional SC was provided with a CD containing all the prepared training materials, handouts, own work and developed materials, as well as photographs taken during each workshop for further dissemination to districts. Although all workshop participants met in their regional and district SCs on a fairly regular basis and participants were encouraged to utilise the resources provided at the workshops, they were also encouraged to contact the facilitators between the contact opportunities. However, the high cost of international calls and lack of general internet access limited this kind of individual interaction with the facilitators in South Africa. Contact was therefore maintained with the SC members by sending regular text messages via their personal mobile phones. These messages were designed as reminders of important KMC practice aspects and as a means of encouraging members to continue with their KMC endeavours.

### Assessing progress in the implementation of KMC

**Figure 2:**
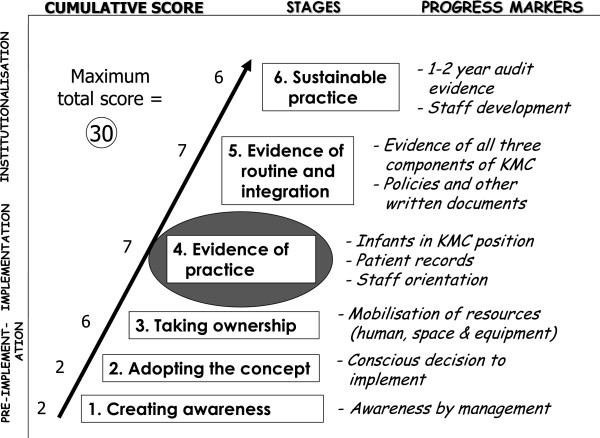
**Model for progress monitoring**[28].

Phase III of *KMC Ghana* included the monitoring of implementation progress based on a stages-of-change model (see Figure 2) [28]. The model represents a stepwise implementation strategy, beginning with ‘pre-implementation’ activities like sensitisation, training, and the adoption of the KMC concept. ‘Implementation’ activities include the mobilisation of resources and the first ensuing ‘evidence of KMC practice.’ This is followed by the ‘institutionalisation’ of KMC, as demonstrated by the integrated and routine practice of KMC and, finally, ‘sustainable practice,’ which is associated with regular audit and the ability to provide statistics related to KMC. A year after *KMC Ghana* commenced, healthcare facilities were expected to be able to demonstrate ‘evidence of KMC practice’ (stage four). A number of quantifiable progress markers had been developed for each stage.

The progress-monitoring model described above was used previously to develop a standardised progress-monitoring instrument to collect data for each hospital [28]. Some items in the instrument contribute to a progress score out of a total of 30 points. A health facility must score 10 points or more to reach the fourth level of progress, namely ‘evidence of practice.’ A score is calculated for individual hospitals and health centres, but can also be given as an average score for a group of institutions. It is also possible to visually represent individual scores on the progress-monitoring model to provide feedback to a health authority on the progress in a particular geographical area or for all institutions targeted in a specific intervention. A weighted score is calculated for each step of the progress-monitoring model (see Figure 2) [28]. After obtaining the initial scores, a comparison was also drawn between the scores of hospitals with baby-friendly status designated or re-designated within the five years prior to the intervention and the scores for the rest of the hospitals, using the chi square (with the Yates correction) and Fisher’s exact test.

A team of three to four local assessors per region were trained in the application of the progress-monitoring tool and the same team visited all the hospitals in a region together, with each team member completing the items in the tool independently. After each visit all items were checked for inter-rater differences, and the team reached consensus on each response for which a discrepancy had been identified. All 38 hospitals were visited according to the planned schedule, and representatives from districts without a hospital joined in the visit to the nearest district hospital. Assessors left a written report with their findings, impressions, and recommendations before leaving the hospital.

Indicators for measuring the impact of KMC on mortality and morbidity were not included as part of the formal evaluation, because only a year had elapsed since the introduction of KMC, and this was considered too short a time in which to measure the long-term outcome. Furthermore, detailed data distinguishing between different categories of infants with low birth weights were not collected by the health system at the time of the intervention.

### Process evaluation

Evaluation of the process of implementing the project consisted mainly of the collection of qualitative data at the points of contact between facilitators and participants and through correspondence with key role-players. All training sessions were evaluated anonymously in different formats that included the traditional satisfaction questionnaire (with open-ended questions and Yes/No or Likert-scale type questions), group drawings, and quantitative and qualitative self-reflection on learning achieved. Halfway through a training workshop, recommendations were made to facilitators on what to improve. The assessors completed a process questionnaire on their experiences during the final assessment in Phase III.

During Phases II and III, steering committee members were required to give formal feedback in the form of presentations on their achievements and challenges. They also carried out regular analyses of strengths and challenges and their feedback on problems and how they had been solved at the local level was documented. Regular SC reports to the Regional Directors of the GHS and facilitator reports to the National Director of the GHS captured the findings on specific phases of the initiative. The facilitators also reflected and reported regularly on their evaluation of the workshops in terms of outcomes and objectives achieved and on their impressions on achievements and challenges.

## Results

Thirty-eight hospitals participated in *KMC Ghana*—33 district hospitals, four regional hospitals, and one teaching hospital. A total of 167 health worker delegates attended one of the six introductory KMC workshops in Phase I. This number corresponded more of less to three delegates from each of the 51 districts targeted in *KMC Ghana*. Fifty-seven of these delegates also attended one of the initial workshops for regional SC members. In Phase II, 59 regional SC members attended one of the four advanced capacity building workshops.

### Process evaluation

Because the wealth of findings from the process evaluation warrants a separate report, only some of the main issues are mentioned here. The evaluation of all training workshops was generally positive with regard to quality and usefulness. Various technical and logistical problems in some of the regions may have impacted on the quality of training (*e.g.*, power failures, problems with audiovisual equipment, lack of printing facilities). Participants appreciated the interactive ‘natural learning environment,’ which they found ‘motivating,’ and they mostly perceived the content of the planned activities as ‘well explained’ or ‘clearly presented.’ Delays in starting sometimes forced facilitators to adapt the programme and shorten some sessions; some participants experienced these shorter sessions as ‘rushed,’ with the material insufficiently explained.

Strengths and challenges were associated with a variety of topics, and what was considered to be a strong point in one health facility or district may have been considered an obstacle in another. The main themes identified were: regional or district infrastructure (roads and transport); management (level of support and interest); resources (financial, material, human); collaboration and team work; the hospital setting (space and equipment); staffing (general level of training, numbers and shortages, attitudes, KMC sensitisation and education); educational opportunities for promoting KMC (in health facilities, communities and districts); mothers (acceptance of KMC, compliance); and the community (cultural and religious beliefs, level of support for mothers with LBW infants).

Constraints for the facilitators were also identified and pertained mainly to logistical issues. As this was a huge scale-up project, many communication mechanisms had to be created in order to facilitate the smooth running of all the activities. In cases where notifications of workshop dates and venues were sent out fairly late, some participants found it difficult to arrive on time. Having regularly updated, written plans of action, not only for the work of the facilitators, but also for the SCs at regional, district and facility level, was helpful in monitoring the flow of activities and identifying potential snags that might affect the execution of the project and the implementation of KMC.

Although the use of cell phone messages to encourage SC members in their KMC implementation work was not formally evaluated, members from all regions expressed their appreciation for this kind of support, and there were 32 responses with feedback on progress with implementation, for example: ‘Glad for your constant reminders. We are on it’; ‘KMC is working. Expecting to hear more from u’; ‘KMC steering comm. meeting in progress. We are having useful discussions. Reg. director is in attendance.’ Occasionally an SC member initiated a phone call, mostly to report on achievements.

### Implementation scores

**Figure 3:**
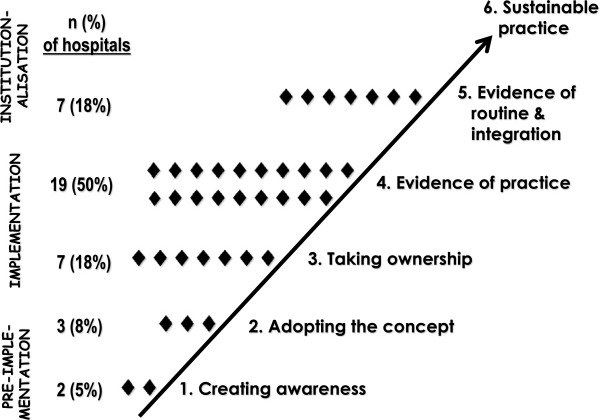
KMC implementation progress in the four regions.

The results of scoring implementation progress are graphically depicted in Figure 3. Hospital scores ranged from 1.55 to 20.69, with a mean score of 12.07 and a median of 12.42. Twelve of the 38 hospitals visited had not reached the level of ‘evidence of practice’ (32 %). Two hospitals were still at the first level of ‘creating awareness’ (5 %); three were at the second level of ‘conscious decision to implement’ (8 %); and seven at the third level of ‘taking ownership’ (18 %). Twenty-six hospitals had reached at least the level of ‘evidence of practice’ (68 %), with seven of these already having reached the level of ‘evidence of routine and institutionalised practice’ (18 % of the total). Two of the hospitals that had not reached the level of ‘evidence of practice’ had made all the necessary preparations in order to be able to practice KMC; one had not delivered any LBW infants at their facility by the time of the visit, while the other had no space and all mothers and infants had to be discharged within two hours after birth. In at least one hospital, cultural factors in the community were playing a role in resistance to KMC. Most of the hospitals that experienced difficulties in implementing KMC were in remote, rural districts where advocacy activities were difficult to organise and where it was not possible to access all the potentially available information and support.

Scores were broken down further on the basis of the baby-friendly status of hospitals. Of the 38 hospitals visited, 26 were designated as baby-friendly hospitals (68 %). Sixteen hospitals had obtained their baby-friendly accreditation or re-accreditation within the past five years (42 % of the total) and scored an average of 14.64 out of a possible 30 points. Nine hospitals had received baby-friendly status more than five years previously (24 %) and had not been re-assessed since that time. They had a mean score of 10.40. The 13 hospitals that did not have baby-friendly status (34 %) scored 10.07. All seven hospitals that scored on the level of ‘evidence of routine and institutionalised practice’ had been designated as baby-friendly between 2004 and 2009. This is statistically highly significant (p < 0.0005) when comparing the group of hospitals with designated baby-friendly status in the past five years with those hospitals that had not been re-assessed for baby-friendliness in the past five years and with those that had never received baby-friendly status.

## Discussion

The progress-monitoring results showed that it was possible to scale up KMC in the four regions in Ghana. The mean and median progress scores achieved in the *KMC Ghana* initiative were slightly lower than those achieved in the South African studies. The mean progress score for the Ghana initiative was 12.07 (SD ± 5.06) compared to 13.43 (SD ± 5.05) and 14.94 (SD ± 5.04) (unpublished data) in the two South African studies. Caution should be exercised not to read too much into these comparisons. The South African studies were randomised trials measuring different outreach strategies and the scores given above are combined scores for the two strategies utilised in each trial.

Although the outreach strategies used in *KMC Ghana* were adapted from the South African model, the lower score does not necessarily mean that the outreach was less effective or less successful. The score could be explained by the fact that Ghana was ‘virgin territory’ with virtually no institution or health worker having had previous exposure to KMC, that it takes time to become familiar with new practices, and that more time would be necessary to master certain practices. South Africa’s refined audit systems were in place [29], and KMC had already been introduced in the mid-1990s [30], enabling hands-on training at hospitals with KMC services. Many health workers were therefore aware of KMC by the time the interventions took place [16], some hospitals had prior experience with KMC [16], and KMC had already been recommended as the policy of choice in the routine care of LBW infants [31]. Furthermore, health systems in the two countries are also organised differently. Skilled birth attendance is sometimes used as a marker of health system access, and countries are divided into three categories of ‘health system context’: low (attendance at birth less than 30 %); middle (30-60 %); and high (greater than 60 %). South Africa is considered to fall into the category of high health system context in Africa, whereas Ghana is considered to fall into the category of middle health system context [32].

The mean score of 14.60 for Ghanaian hospitals that had achieved baby-friendly status in the five years prior to the KMC intervention compares well with the mean South African scores. It could be argued that the preparation and processes applied by hospitals in order to become baby-friendly had a positive influence on their ability to implement KMC.

According to the updated Cochrane systematic review [4], KMC was found to reduce mortality at discharge or 40 to 41 weeks’ postmenstrual age and at latest follow-up when compared to conventional neonatal care. It was also found to reduce nosocomial or severe infection, hypothermia, severe illness, lower respiratory tract disease, and length of hospital stay [4]. The reviewers conclude: ‘Since the control group in studies evaluating continuous KMC was in incubators or radiant warmers, the potential beneficial effects of KMC on morbidity and mortality of LBW infants would be expected to be greatest in settings in which conventional neonatal care is unavailable’ [4].

When *KMC Ghana* was initiated, the under-five, infant and neonatal mortality rates were estimated to be around 115, 73, and 43 per 1,000 live births, respectively [13]. Since then, there has been a further decline to 80, 50, and 30 per 1,000 live births, respectively [33]. These declines cannot be directly attributed to the *KMC Ghana* initiative, because other newborn care initiatives are also being undertaken. However, it appears likely that the introduction of KMC would have some overall beneficial effect, because most of the hospitals included in the project did not have incubators.

Any future audit system should include weight categories that can provide a breakdown of birth weight in 500 gram increments. Such a system would provide more reliable data because it would be able to distinguish between the morbidity and mortality outcomes of LBW infants and very LBW infants, who are at greater risk.

## Conclusions

One KMC research area identified by Victora *et al*. [34] is the need to bring KMC closer to the population. According to these authors, in most low- and middle-income countries KMC implementation started at a teaching or other tertiary hospital without expanding to district hospitals. *KMC Ghana* illustrated that a roll-out of KMC was possible in the absence of an established centre of excellence at a teaching hospital at the time of implementation. It is essential, however, to identify and develop regional and district hospitals as centres of excellence where health workers can receive the necessary orientation and training to initiate KMC or reinforce current practices.

The findings from *KMC Ghana*’s process evaluation demonstrated how findings from two randomised trials regarding successful outreach for the implementation of KMC could be translated to a different context by making appropriate adaptations to suit different settings and improve acceptability of a scale-up initiative. When international facilitators are used, sufficient experience [21] and a mix of expertise is needed to demonstrate the importance of a multidisciplinary team approach in KMC implementation and the need for sufficient prior familiarisation with the context.

As has been found in other large-scale KMC initiatives, sustained support for the implementers on the ground is important for the institutionalisation of KMC in healthcare facilities. More research is also needed on approaches to integrate KMC implementation with other programmes such as Essential Newborn Care (ENC), the Baby-friendly Hospital Initiative (BFHI), the Integrated Management of Neonatal and Childhood Illnesses (IMNCI), and High Impact Rapid Delivery (HIRD) and to strengthen supportive supervision that will help to sustain momentum for KMC practice.

Some regions suggested that the outreach and progress-monitoring model followed in the *KMC Ghana* initiative could be used to plan other country-wide monitoring exercises by identifying relevant indicators (progress markers) for each step of the stages-of-change model and devising a specific progress-monitoring measuring tool and score for each project. The progress monitors were particularly impressed by the way in which ‘no one could hide’ anything when this particular approach was used.

## Competing interests

The authors participating in this initiative received their normal salaries for the duration of the project and did not benefit from any additional incentives. *Per diems* were paid according to the rules of the funding agency.

## Authors’ contributions

All authors were involved in the conceptualisation of the initiative and execution of the project. A-MB, KD and EvR acted as facilitators, RM managed the project and GQA, JKA-W, MD, AN and AT supervised the project on behalf of the Ghana Health Service. All authors contributed to the draft article and approved the final version. All authors read and approved the final manuscript.
